# Involvement of Secondary Induced Thrombus on Hemorrhage Induced by Both Delayed Recanalization and Delayed t-PA Treatment in Murine Ischemic Stroke Models

**DOI:** 10.3390/biomedicines14020308

**Published:** 2026-01-29

**Authors:** Yuhki Moriike, Yumeta Nakano, Yasuki Matano, Yasuhiro Suzuki, Kazuo Umemura, Nobuo Nagai

**Affiliations:** 1 Department of Animal Physiology, Faculty of Bioscience, Nagahama Institute of Bio-Science and Technology, Tamura 1266, Nagahama 526-0829, Shiga, Japan; 2 Department of Pharmacology, Faculty of Medicine, Hamamatsu University School of Medicine, Handa-cho 1-20-1, Hamamtsu 431-3192, Shizuoka, Japan; 3 School of Pharmaceutical Sciences, Ohu University, Tomitasankakudo 31, Kohriyama 963-8611, Fukushima, Japan

**Keywords:** endovascular therapy, glycocalyx, hemorrhage, ischemic stroke, tissue-type plasminogen activator

## Abstract

**Background**: In the treatment of ischemic stroke, both tissue-type plasminogen activator (t-PA) thrombolytic and endovascular therapy are employed; however, delayed intervention with these therapies increases the risk of hemorrhage. Hemorrhage associated with delayed t-PA treatment involves the activation of plasmin and matrix metalloproteinases (MMPs); however, the detailed mechanisms underlying I/R activation remain unclear. **Objectives**: This study examined the effects of delayed recanalization on ischemic stroke in a permanent middle cerebral artery (MCA) occlusion (MCA-O) model, and a novel MCA ischemia/reperfusion (I/R) model: 2-h ischemia followed by reperfusion (I/R 2 h), and 4.5-h ischemia followed by reperfusion (I/R 4.5 h). Secondary induced thrombus (SIT) formation, hemorrhage, MMP activity, MMP-9 immunoreactivity, and tomato lectin (TL) staining, as well as the effects of t-PA and heparin treatment were evaluated. **Results**: SIT formed within 1 h after reperfusion in the I/R 4.5 h model only, while t-PA or heparin treatment reduced SIT formation. Hemorrhage increased with or without t-PA administration in the I/R 4.5 h model, but it was suppressed by heparin pretreatment. MMP activity and MMP-9 immunoreactivity were localized to the SIT. Additionally, a negative staining area for TL was observed in the damaged area, where SIT formed in the I/R 4.5 h model. **Conclusions**: These results suggest that delayed recanalization induces SIT via glycocalyx degradation, leading to hemorrhage via plasmin/MMP-9 activation by endogenous and exogenous t-PA-mediated fibrinolysis in novel murine models of ischemic stroke. Furthermore, inhibition of SIT formation is beneficial for suppressing hemorrhages associated with delayed recanalization after endovascular or t-PA therapy.

## 1. Introduction

Ischemic stroke is caused by the occlusion of cerebral blood vessels by thrombi, leading to ischemia and subsequent necrosis of the brain tissue. It accounts for about 60% of cerebrovascular disorders and affects approximately 12 million people yearly [1]. Recombinant tissue-type plasminogen activator (t-PA) is used as a thrombolytic agent to treat ischemic stroke [2]. However, delayed recanalization following t-PA therapy increases the risk of hemorrhage [2]. Consequently, recombinant t-PA treatment is restricted to within 4.5 h of stroke onset [3], resulting in a low clinical utilization rate of approximately 10%. Previous studies have reported that the increased hemorrhagic risk of t-PA treatment is due to the activation of matrix metalloproteinases (MMPs) 3 and 9 [4,5]; however, the detailed molecular mechanism of MMP activation remains unclear. In addition, although endovascular therapy (EVT), which removes the thrombus using an intra-arterial catheter to restore blood flow, is widely performed in ischemic stroke, delayed treatment also increases the risk of hemorrhage [6]. Therefore, to clarify the mechanisms of hemorrhage on delayed EVT and thrombolytic therapy, the MMP activation mechanism is especially beneficial for clinical treatment.

Reperfusion after vascular occlusion provides beneficial effects but also triggers inflammation via secondary reactions through immune cell infiltration [7] and platelet activation [8], thereby exacerbating prognosis after cerebral infarction. Furthermore, it has been suggested that reperfusion following a primary occlusive thrombus leads to the formation of a secondary induced thrombus (SIT) [9,10], exacerbating cerebral infarction [11,12].

In this study, SIT formation was examined in a permanent middle cerebral artery (MCA) occlusion (MCA-O) [13] and an MCA ischemia/reperfusion (I/R) model, which is newly established by modification of the MCA-O model. No or few SITs were induced in the MCA-O and 2 h ischemia/reperfusion (I/R 2 h) models, respectively, whereas remarkable SIT was induced in the 4.5 h ischemia/reperfusion (I/R 4.5 h) model. Hemorrhage with or without t-PA treatment was also evaluated using these models.

## 2. Materials and Methods

### 2.1. Animals

All animal experiments were performed in accordance with the recommendations of the Guide for the Care and Use of Laboratory Animals of the Japan Society for the Promotion of Science. Ethical approval was obtained in accordance with the institutional rules for the care and use of laboratory animals of the Nagahama Institute of Bio-Science and Technology (Approval No. 017).

Male BALB/c mice (Japan SLC Inc., Hamamatsu, Japan) weighing 22–27 g and aged 8–12 weeks (n = 159 in total) were used in the study. They were housed in 23 +/− 1 °C environment with a 12 h light and 12 h dark cycle. The number of animals in each experiment is indicated in figure legends. Experimental treatments for each animal were randomized and their order on individual days were changed in order to minimize confounders. The number of animals in each experiment were chosen from previous experiments in which prior power analysis was performed [14]. The humane endpoint was the absence of spontaneous movement and severe bleeding after emergence from anesthesia, but no animals were dropped in the experiments.

### 2.2. Whole Experimental Setup and Ischemic Stroke Models

The whole experimental setup is described in Supplementary Figure S1.

Brain damage is induced by the permanent occlusion of the MCA [13] or by reperfusion following ligation. Mice were briefly anesthetized with 5% isoflurane and maintained on 2.5%. The skull was exposed by incising the left temporal muscles and retracting the overlying muscles. A 1-mm window was created directly over the MCA, immediately above the zygomatic arch, using a surgical drill (TKG U-Hobby Standard Set UHB-1; Tokyo Glass Instruments, Tokyo, Japan). The exposed MCA was ligated using a 10-0 nylon suture (Johnson & Johnson K.K., Tokyo, Japan) to induce permanent occlusion (MCA-O model). For reperfusion, the MCA was occluded using sterilized mouse whiskers. The scalp skin incision was sutured in place with a mouse whisker, and the mouse was awakened. At 2 h (I/R 2 h model) or 4.5 h (I/R 4.5 h model) after occlusion, the skin at the surgical site was reopened under anesthesia, the whisker was removed, and the suture occluding the MCA was excised to restore MCA patency. The skin incision was sutured again.

### 2.3. Blood Flow Measurement

Cerebral blood flow was measured via a window created over the MCA using a laser Doppler flowmeter (UNIQUE MEDICAL, Tokyo, Japan). Measurements were taken before and immediately after MCA occlusion at 5, 15, and 30 min after reperfusion, and at 24 h after MCA ligation. For each time point, the average of 4 blood flow measurements was calculated and expressed as a percentage of the pre-occlusion value.

### 2.4. Visualization of SIT, Glycocalyx, and Vascular Permeability Following Cerebral Infarction

The SIT formed after cerebral infarction was visualized following intravenous administration of Fibrinogen (Fbg)-FITC (Innovation Research, Novi, MI, USA) after MCA occlusion. In the MCA-O model, 8 mg/kg of Fbg-FITC was administered intravenously 1 h after occlusion, whereas in the I/R model, it was administered immediately before reperfusion. Additionally, Tomatolectin-649 (TL; Vector Laboratories, Burlingame, CA, USA), which binds to vascular glycocalyx, was administered intravenously at 2 µL/g 1 h before euthanasia. Furthermore, 50 mg/kg of dextran-TRITC (Dex; TdB Labs, Uppsala, Sweden), an indicator of vascular permeability, was also administered one hour before euthanasia.

### 2.5. Administration of t-PA, Heparin, and Aprotinin

To examine the contribution of SIT to hemorrhage, 10 mg/kg of t-PA (Actibacin; Kyowa KIRIN, Tokyo, Japan) was administered intravenously immediately before reperfusion. In addition, to investigate the effect of inhibiting SIT formation, heparin (500 U/kg; Mochida Pharmaceutical, Tokyo, Japan) was administered intravenously immediately before reperfusion. To examine the effect of the endogenous fibrinolytic system on thrombolysis, aprotinin (4 mg/kg; FUJIFILM Wako Pure Chemical Corporation, Tokyo, Japan) was administered intravenously immediately before reperfusion. These drugs were administered through the tail vein.

### 2.6. Measurement of the Amount of SIT and TL Negative Area

After Fbg-FITC and TL administration, the mice were euthanized by deep anesthesia with Ravonal (Nipuro Co., Ltd., Osaka, Japan), and the brains were collected. Coronal brain sections were prepared and observed using a confocal microscope (FV4000, Evident Corporation, Tokyo, Japan). For the SIT, the number and area of FITC-positive signals in each section were measured. The size of the TL-negative region was quantified using ImageJ (v1.54b, National Institutes of Health, Bethesda, MD, USA) after photographing the slices.

### 2.7. Quantification of Damage Size and Bleeding Volume

The cerebral infarction area was assessed by 2,3,5-triphenyltetrazolium chloride (TTC; Sigma-Aldrich, St. Louis, MO, USA) staining of 1 mm-thick brain sections at 1.5 mm, 0.5 mm, −0.5 mm, −1.5 mm, −2.5 mm, and −3.5 mm from Bregma in brain atlas [15]. Then it was followed by image analysis as previously described [13]. Bleeding volume was measured by imaging analysis of the brain section center and Drabkin’s reagent assessment, as described previously [16].

### 2.8. MMP Activity

To visualize matrix MMP activity, 100 µL of MMPsense680 (PerkinElmer Japan G.K., Tokyo, Japan) was administered intravenously. One hour after administration, the mice were euthanized, and 1 mm-thick brain slices were prepared and observed using a confocal microscope (FV4000, Evident Corporation, Tokyo, Japan).

### 2.9. Immunohistochemistry

The localization of CD31, MMP-3, and MMP-9 was assessed by immunohistochemistry. Eight hours after MCA ligation for CD31, and twenty-four hours after MCA ligation for MMP-3 and MMP-9, mice were fixed with 4% paraform aldehyde under deep anesthesia, and 8 µm-thick frozen sections were prepared. The sections were treated with anti-CD31 (Bioss, Boston, MA, USA), anti-MMP-3 (Santa Cruz Biotechnology, Dallas, TX, USA), or anti-MMP-9 (Abcam Plc, Cambridge, UK) antibodies. They were then treated with a biotin-conjugated secondary antibody specific to each primary antibody, followed by a fluorescently labeled Cy3-conjugate streptavidin (AAT Bioquest, Pleasanton, CA, USA). The sections were observed under a fluorescence microscope (BX53; OLYMPUS, Tokyo, Japan).

### 2.10. Statistical Analysis

Statistical analyses were performed using ANOVA, followed by Fisher’s PLSD or Dunnett’s test for post hoc analysis, or Student’s *t*-test for comparisons between groups; *p*-values less than 0.05 were considered statistically significant.

## 3. Results

### 3.1. Blood Flow and Lesion Size in MCA-O and I/R Models

**Figure 1:**
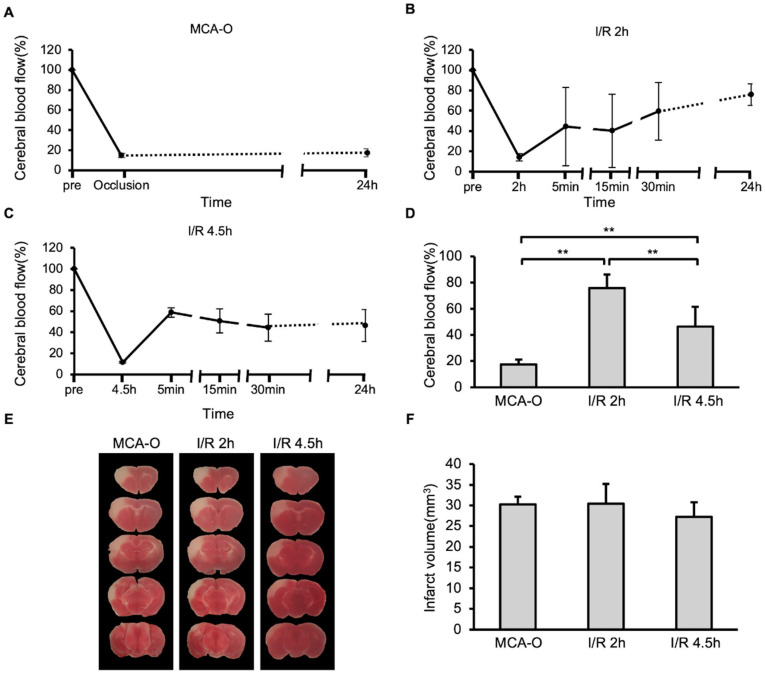
Blood flow and damage size in stroke models. (**A**–**C**): Temporal changes of cerebral blood flow in models of MCA-O (**A**), I/R 2 h (**B**), and I/R 4.5 h (**C**). (**D**): Cerebral blood flow at 24 h. Data represent mean and SD. Each group contains 6 mice. **: *p* < 0.01. (**E**): Photographs of the TTC-stained brain sections of MCA-O, I/R 2 h, and I/R 4.5 h models at 24 h post-cerebral infarction induction. (**F**): Damage size in TTC stained sections at 24 h. Data represent mean and SD.

In the MCA-O model, blood flow decreased following vascular occlusion to approximately 15% of its pre-occlusion level, and this persisted even after 24 h (Figure 1A). In the MCA-I/R model, blood flow also decreased to approximately 15% immediately after ligation but recovered to approximately 50% after reperfusion (Figure 1B,C). At 24 h after MCA ligation, blood flow recovered to 80% of its pre-occlusion level in the I/R 2 h model. In contrast, it recovered to below 50% in the I/R 4.5 h model, which was significantly lower than in the I/R 2 h model (Figure 1D). TTC staining revealed no significant differences in infarct size between groups (Figure 1E,F). These results indicate that neuronal cell damage in the MCA territory was maximally induced by 2 h of ischemia.

### 3.2. SIT and Vascular Permeability

**Figure 2:**
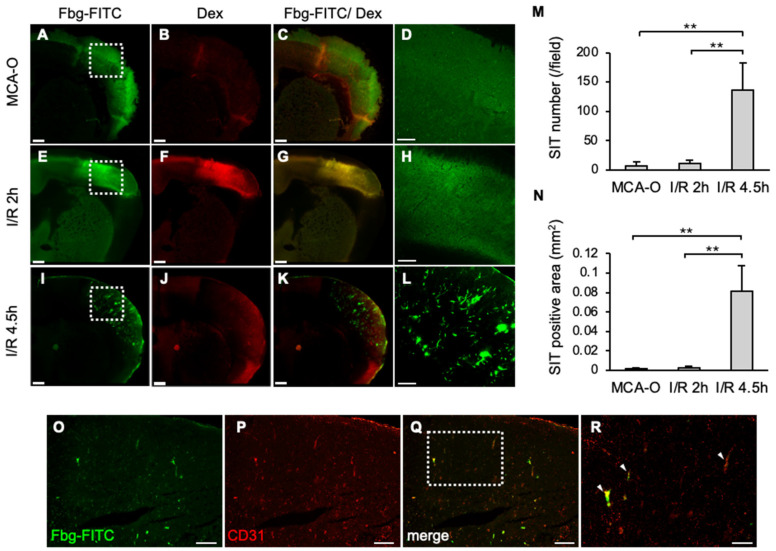
SIT and dextran leakage at 8 h after MCA ligation in each model. (**A**–**L**): Photographs of coronal sections of mice in MCA-O (**A**–**D**), I/R 2 h (**E**–**H**), and I/R 4.5 h (**I**–**L**). Images of Fbg-FITC (**A**,**E**,**I**), Dextran (**B**,**F**,**J**), and their merged images (**C**,**G**,**K**) are shown, respectively. Higher magnified image of squares in (**A**,**E**,**I**) are shown in (**D**,**H**,**L**). Bars represent 500 µm in (**A**–**C**,**E**–**G**,**I**–**K**), and 200 µm in (**D**,**H**,**L**). (**M**,**N**): Quantified data of the number (**M**) and the area (**N**) of Fbg-FITC. Data represent mean and SD. Each group contains 4 mice. **: *p* < 0.01. (**O**–**R**): Photographs of mouse in I/R 4.5 h in Fbg-FITC (**O**), CD31 (**P**) and their merged image (**Q**) are shown. Higher magnified images of squares in (**Q**) are shown in (**R**). Arrows indicate merged signal of Fbg-FITC and CD31 immunoreactivity. Bars represent 100 µm in (**O**–**Q**), and 50 µm in (**R**). Comparable results were observed in IHC for CD31 (n = 3).

At 8 h, Fbg-FITC-positive areas were observed in sections from each model, coinciding with dextran-positive areas of vascular permeability in MCA-O and I/R 2 h (Figure 2A–C,E–G). These results suggested that vascular permeability increased due to ischemia or ischemia/reperfusion stress, and that Fbg-FITC leaked into the area. In the I/R 4.5 h model, remarkable numbers of Fbg-positive vascular-like structures were observed within the area (Figure 2I,L), whereas no or few Fbg-positive signals were in the MCA-O (Figure 2A,D) and I/R 2 h models, respectively (Figure 2E,H). These Fbg-positive signals were distributed with a positive signal of CD31, an endothelial cell marker, indicating that these Fbg-positive vascular-like structures were SIT. The quantified results showed that both the number and area of the SIT were markedly increased only in the I/R 4.5 h model (Figure 2M,N), indicating that delayed reperfusion induces SIT in the ischemic area.

### 3.3. Effects of t-PA and Heparin Administration on SIT Formation

**Figure 3:**
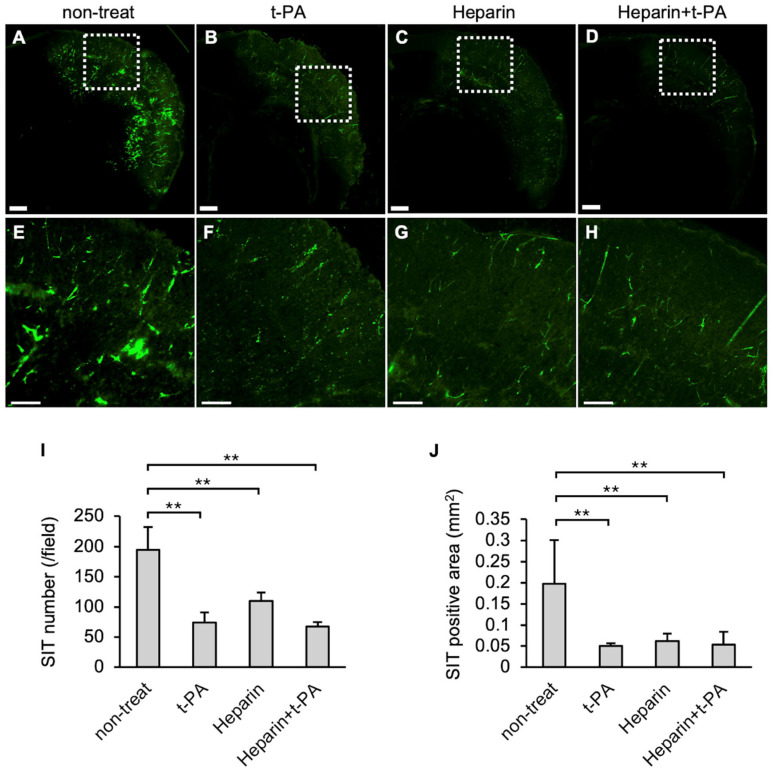
Effect of t-PA and heparin on the SIT in I/R 4.5 h model at 5.5 h after MCA ligation. FITC-fluorescence photographs of coronal sections of mice in non-treat (**A**,**E**), t-PA (**B**,**F**), heparin (**C**,**G**), and heparin + t-PA (**D**,**H**). Higher magnified image of squares in (**A**–**D**) were shown in (**E**–**H**), respectively. Bars represent 500 µm in (**A**–**D**), and 200 µm in (**E**–**H**). (**I**,**J**): Quantified data of the number (**I**) and the area (**J**) of Fbg-FITC. Data represent mean and SD. Each group contains 4 to 6 mice. **: *p* < 0.01.

The effects of t-PA and heparin on SIT formation in the I/R 4.5 h model were examined. The numbers and area of FITC-positive vessel-like structures were reduced in the t-PA-treated group (Figure 3B,F) compared with the non-treated group (Figure 3A,E), indicating that t-PA treatment accelerated SIT degradation. The heparin-treated group also showed a significant decrease in SIT formation (Figure 3C,G). Furthermore, the heparin-and subsequent t-PA-treated groups exhibited a significant reduction in the SIT (Figure 3D,H) compared to the non-treated group. The numbers and area of the SIT were markedly reduced following treatment with t-PA, heparin, or heparin followed by t-PA, compared with the non-treated group (Figure 3I,J).

### 3.4. Blood Flow and Infarct Size Following Administration of t-PA and/or Heparin in an I/R 4.5 h Model

**Figure 4:**
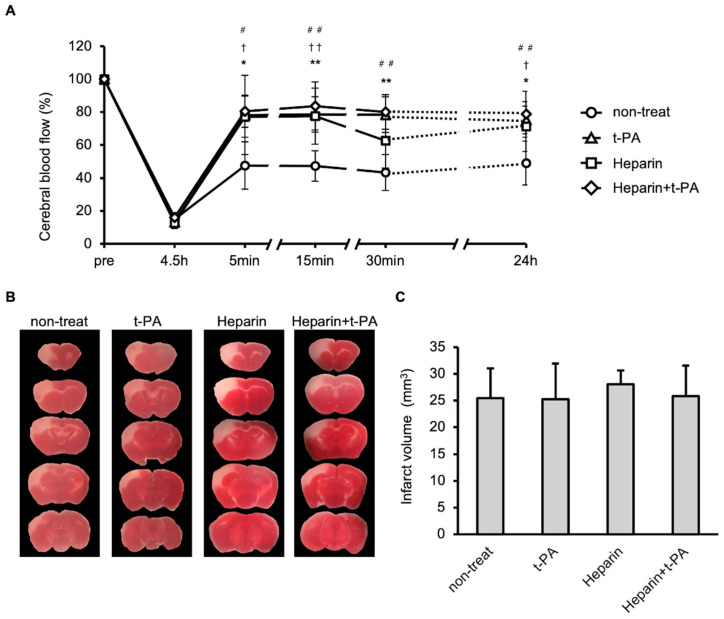
Effects of t-PA and heparin on the SIT formation and blood flow at I/R 4.5 h. (**A**): Temporal changes of blood flow in non-treat, t-PA, heparin, and heparin + t-PA. Data represent mean and SD. *: *p* < 0.05, **: *p* < 0.01 t-PA vs. non-treat, †: *p* < 0.05, ††: *p* < 0.01 heparin vs. non-treat, #: *p* < 0.05, ##: *p* < 0.01 heparin + t-PA vs. non-treat. (**B**): TTC-stained brain sections at 24 h after MCA ligation. (**C**): Quantified results of damage size in TTC stained sections. Data represent mean and SD. Each group contains 6 mice.

The effects of t-PA and heparin on blood flow and infarct size were studied using the I/R 4.5 h model. Compared with the non-treated group, treatment with t-PA, heparin, and heparin + t-PA groups restored blood flow at 24 h (approximately 50% in the non-treated group vs. 70–90% in other groups, Figure 4A). However, t-PA, heparin, and heparin with t-PA treatment did not affect the infarct volume at 24 h (Figure 4B,C).

### 3.5. Hemorrhage

**Figure 5:**
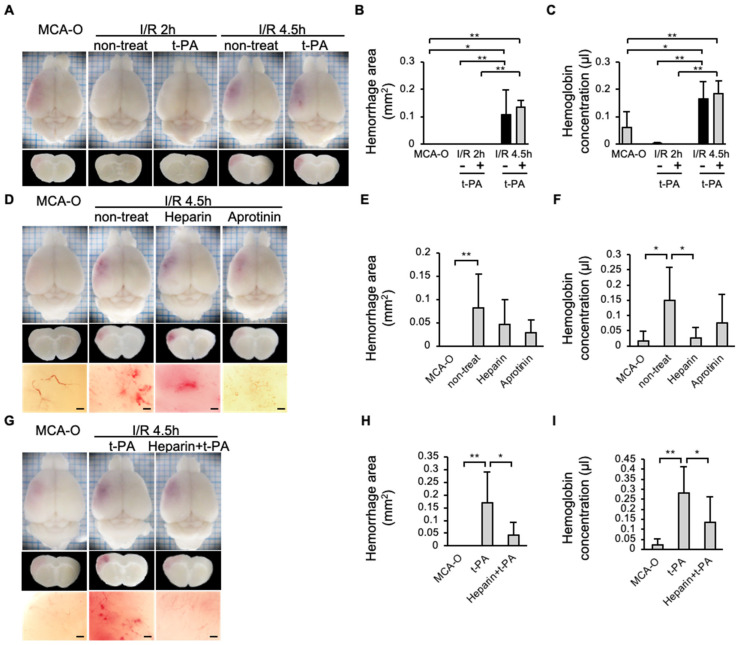
Effects of t-PA, heparin, and aprotinin on hemorrhage at 24 h after cerebral infarction in each model. (**A**–**C**): Photographs of the brain surface and sections in models of MCA-O, I/R 2 h, and I/R 4.5 h with or without t-PA treatment (**A**), and quantified results in area (**B**) and amount (**C**) of hemorrhage. Each group consists of 5 mice. (**D**–**F**): Photographs of the brain surface and sections in the MCA-O and non-treat, treatment of heparin and aprotinin in I/R 4.5 h (**D**), and quantified results in area (**E**) and amount (**F**) of hemorrhage. Each group consists of 6 mice. (**G**–**I**): Photographs of the brain surface and sections in the MCA-O and treatment of t-PA and heparin + t-PA in I/R 4.5 h (**G**) and quantified results in area (**H**) and amount (**I**) of hemorrhage. Each group consists of 5 mice. Data represent mean and SD. *: *p* < 0.05, **: *p* < 0.01. Bars represent 100 µm in (**D**,**G**).

In the MCA-O or I/R 2 h groups, no hemorrhage was observed 24 h after MCA ligation, even with administration of t-PA in the I/R 2 h model. In contrast, the hemorrhage area increased in the I/R 4.5 h model compared to the MCA-O or I/R 2 h model (Figure 5A,C). The type of hemorrhage observed in the current study was petechiae (Figure 5D,G). In addition, in the I/R 4.5 h model, hemorrhage was reduced in the heparin-treated group compared with the non-treatment group (Figure 5E,F). Although the amount of hemorrhage in the aprotinin-treated group did not differ from that in the untreated group (Figure 5E,F), the petechiae hemorrhages observed in the untreated group disappeared (Figure 5D). Furthermore, in the heparin + t-PA-treated group, hemorrhage was significantly reduced compared with the t-PA-treated group (Figure 5H,I).

### 3.6. MMP Activity and Immunohistochemistry of MMP-3 and MMP-9

**Figure 6:**
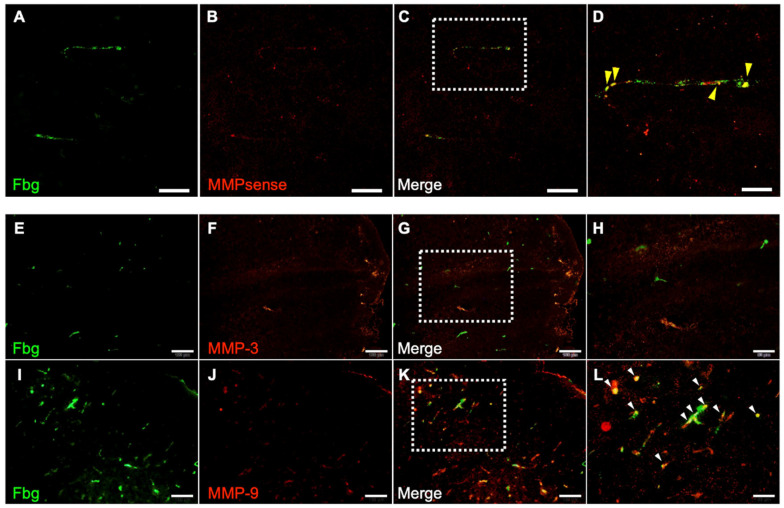
Distribution of MMP activity and immunoreactivity of MMP-3 and MMP-9. Photographs of sections in damaged area in I/R 4.5 h model at 5.5 h after MCA ligation. (**A**–**D**): Fbg-FITC (green in (**A**)), MMPsense (red in (**B**)), and their merged image (**C**) in same section. Higher magnified image of a square in (**C**) is also shown in (**D**). Arrows indicate merged signal of Fbg-FITC and MMPsense. Bars represent 50 µm in (**A**–**C**), and 20 µm in (**D**). n = 4 for this experiment. (**E**–**L**): Fbg-FITC (**E**,**I**), MMP-3 (**F**) or MMP-9 (**J**) and their merged image ((**G**,**K**), respectively) are shown. Higher magnified images of squares in (**G**,**K**) are shown in (**H**,**L**), respectively. Arrows indicate merged signal of Fbg-FITC and MMP immunoreactivity. Bars represent 100 µm in (**E**–**G**,**I**–**K**), and 50 µm in (**H**,**L**).

The contribution of MMPs to hemorrhage was examined using the I/R 4.5 h model. One hour after reperfusion, all MMP activities were colocalized with Fbg-FITC-positive SIT in the brain (Figure 6A–D) in the t-PA-treated group. Furthermore, 24 h after MCA ligation, MMP-9 immunoreactivity was distributed in the SIT, whereas MMP-3 immunoreactivity was not observed in the damaged area (Figure 6E–L).

### 3.7. TL Treatment and the SIT

**Figure 7:**
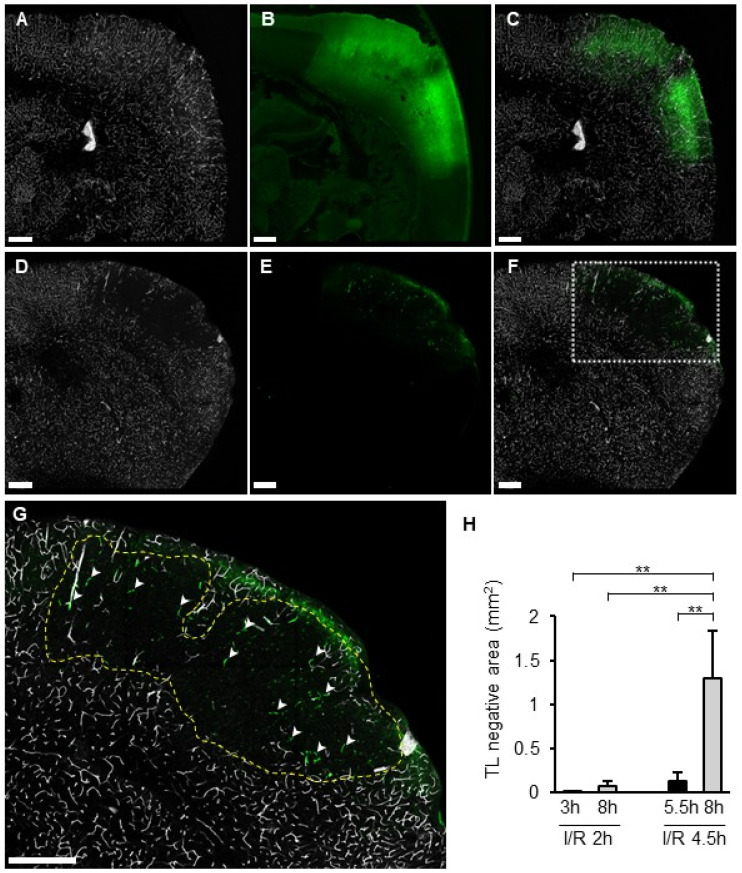
TL deposition and SIT in I/R models. (**A**–**G**): Photographs of coronal sections of mice in I/R 2 h model (**A**–**C**) and I/R 4.5 h model (**D**–**G**) of TL (**A**,**D**), Fbg-FITC (**B**,**E**), and I/R merged images (**C**,**F**). Higher magnified image of a square in (**F**) was shown in (**G**). Dotted line indicates the border of TL negative area. Arrowheads indicate SIT. (**H**): Quantified data of the percentage of TL negative area in brain section. Each group consists of 4 to 6 mice. In graphs, data represent mean and SD. **: *p* < 0.01.

Distribution of the TL signal and SIT was compared between I/R 2 h and I/R 4.5 h. In the I/R 2 h model, a TL signal was observed in the whole section, including the ischemic area, without SIT (Figure 7A–C). In the I/R 4.5 h model, the TL signal disappeared inside the FITC-leaked area (Figure 7D–F). The size of the TL-negative region was significantly greater in I/R 4.5 h than in I/R 2 h (Figure 7H). In particular, the size of the TL-negative area at 8 h in the I/R 4.5 h model was larger than at 5.5 h (Figure 7H). SIT was observed only in part of the TL-negative region in I/R 4.5 h (Figure 7G).

## 4. Discussion

In this study, we established a novel MCA-I/R model based on the MCA-O model to investigate pathophysiological changes associated with recanalization after cerebral infarction. The intracranial suture model, which is widely used for ischemic stroke research, induces infarction in the MCA territory by placing an embolus inserted via the internal carotid artery at the origin of the MCA [17,18]. Since the ischemia extends to a large region including the striatum and hippocampus in this model and the prolonged occlusion is lethal, the model is often used under conditions of relatively short-term (up to ~3 h) reperfusion. In addition, vessel occlusion must be confirmed with the suture by a flowmeter. Furthermore, the effects of damage of endothelium by insertion of suture and subsequent thrombus formation must be considered. In contrast, since the MCA occlusion is occluded at a relatively distal site in the MCA-I/R model, cerebral infarction is induced only to the cerebral cortex, and therefore is non-lethal. In addition, since occlusion is achieved by ligation, both vessel occlusion and recanalization can be visually confirmed. Furthermore, contact with vasculature is limited to the ligation site; therefore, artificial vascular manipulation can be minimized. Here, we compared infarct size, blood flow, SIT formation, and hemorrhage in three MCA occlusion models (MCA-O, I/R 2 h and I/R 4.5 h). It was found that blood flow after reperfusion recovered to 80% of pre-reperfusion levels in the I/R 2 h group, whereas the I/R 4.5 h group showed approximately 50% recovery at 24 h. In addition, few intravascular depositions of FITC-labeled Fbg were observed in the damaged region in the I/R 2 h model. In contrast, it was detected within the damaged area as a vessel-like structure in the I/R 4.5 h model. These findings indicate that delayed reperfusion (4.5 h) after cerebral infarction induces SIT, thereby reducing blood flow. Although SIT formation following cerebral infarction is widely recognized [8,9,10,11], the conditions underlying its formation remain unclear. The findings of this study indicate that delayed recanalization is associated with the formation of SIT, and that these thrombi inhibit the restoration of blood flow after recanalization.

In addition, the hemorrhage volume significantly increased following delayed reperfusion (I/R, 4.5 h). These findings are consistent with previous reports of increased hemorrhage due to delayed recanalization in human patients [6] and animal experiments [19]. The bleeding observed in the experiments consisted of petechiae (Figure 5D), consistent with a previous report [19]. Following recanalization, the petechiae were suppressed with aprotinin, a plasmin inhibitor, indicating that they resulted from the activation of the endogenous fibrinolytic system. The hemorrhagic volume showed no difference between the MCA-O model and the aprotinin-treated group in the I/R 4.5 h model, likely due to aprotinin’s inhibition of fibrinolytic degradation and blood clot removal in the vasculature.

Furthermore, administration of t-PA immediately before reperfusion in the I/R 4.5 h model significantly increased hemorrhage compared to MCA-O, and even showed a tendency toward increased bleeding compared to non-treatment with t-PA. Further, t-PA is administered just before recanalization of the MCA; therefore, it can be assumed that t-PA treatment mimics the recanalization by degradation of the occlusive clot in ischemic stroke. This result is consistent with previous findings showing that increased bleeding is associated with reperfusion following delayed t-PA administration [2]. However, t-PA administration did not increase hemorrhage in I/R 2 h, when the thrombus was not induced. Considering the increase in hemorrhage by delayed reperfusion alone, it is likely that in delayed t-PA therapy, t-PA dissolves the thrombus of the primary cause of ischemic stroke and induces recanalization, which is thought to lead to the formation of SIT. Then, hemorrhage is induced by activation of the t-PA/plasmin/MMP pathway on the SIT.

Heparin administration prior to reperfusion in the I/R 4.5 h model significantly suppressed SIT formation and reduced exacerbation of hemorrhage caused by reperfusion alone or by reperfusion combined with t-PA administration. These results indicate that SIT induced by delayed recanalization contributes to hemorrhage, and that pharmacological inhibition of SIT formation by heparin or other anticoagulants is useful for reducing bleeding risk during delayed recanalization and delayed t-PA administration.

Delayed administration of t-PA contributes to hemorrhage by degrading the basement membrane of vessels through activation of the t-PA/plasmin/MMP pathway [4,20]. Since plasminogen is hardly activated in solution but is easily activated in fibrin thrombi because of the conformational change from globular to linear [21,22], however, it remains unclear how the t-PA/plasmin/MMP pathway is activated in hemorrhage induced by delayed treatment with t-PA. The MMP activity and MMP-9 immunoreactivity were distributed in the SIT. Given that t-PA administration reduced SIT formation, the t-PA/plasmin system activated within the SIT degraded it, triggering MMP-9 activation. Furthermore, MMP activity was detected only in the thrombi, indicating that it contributes to hemorrhage originating from them. MMP-9 is expressed not only in vascular endothelial cells but also in neutrophils, macrophages, and platelets [23,24,25]. MMP-9 in the SIT is thought to originate from these cells incorporated into the thrombus. Considering that vascular permeability in the ischemic area was already elevated at I/R 2 h, the activated MMP could diffuse to the vascular basement membrane. In summary, the t-PA/plasmin system activates MMP-9 in the SIT, allowing it to reach and degrade the basement membrane, leading to hemorrhage.

In the I/R 2 h model, all vessels within the lesion remained TL-positive even 8 h after MCA occlusion, similar to normal areas. Meanwhile, in the I/R 4.5 h model, the TL-negative area appeared within 5.5 h and expanded to 8 h after MCA occlusion. TL has a protein that binds with high affinity to N-acetylglucosamine oligomers [26] and attaches to the glycocalyx of vascular endothelial cells [27]. Considering that the SIT was formed in the TL-negative region by fibrinogen and the SIT-positive area was smaller than the TL-negative area, the shield with thrombi did not induce TL-negative, but rather the loss of glycocalyx. The glycocalyx is a structure on the surface of the vascular lumen and is composed of proteoglycans, glycosaminoglycans, and sugar chains. Heparan sulfate proteoglycan, one of the major components of the glycocalyx, exhibits antithrombotic properties by containing thrombomodulin [28] and binding with high affinity to antithrombin and heparin cofactor II [29,30,31]. It was reported that the TL-positive glycocalyx decreased following activation of hyaluronidase 1 and heparinase after cerebral ischemia [32]. A 4.5-h ischemia led to glycocalyx degradation, loss of antithrombotic properties, and subsequent thrombus induction. On the other hand, neither TL-negative areas nor SIT were observed in the I/R 2 h model, indicating that 2 h of ischemia was insufficient to degrade the glycocalyx. On the other hand, it still remained a possibility that the appearance of the TL-negative area was not associated with degradation of glycocalyx but other mechanisms including microvascular no-flow by vascular contraction. Another possibility is that the TL-negative area was induced by microvascular no-flow associated with vasoconstriction [33]. Clarifying the details of the glycocalyx’s involvement remains a topic for future investigation.

In the 2 h or I/R 4.5 h models, the cerebral infarction size was comparable at 24 h, indicating that the SIT observed in the I/R 4.5 h model did not worsen cerebral infarction. The ischemic area in a cerebral infarction is divided into the ischemic core, which undergoes rapid necrosis at the center of the lesion following ischemia, and the ischemic penumbra, which is a reversible injury zone. BALB/c, the murine strain used in the current experiments, is known to have a sparse ischemic penumbra and a large ischemic core in the injured area because of its limited collateral circulation [34]. Therefore, the hemorrhage mechanism revealed in this study, mediated by SIT dissolution, is thought to reflect hemorrhage within the ischemic core. However, since it has been reported that SIT exacerbates cerebral infarction in humans and other mouse stroke models [9,10,11,12], it is also conceivable that SIT may be generated in the ischemic penumbra via other mechanisms, necessitating further investigation.

## 5. Conclusions

In the present study, we found that delayed reperfusion led to the formation of SIT within the ischemic core, using a novel murine MCA-I/R model. Inhibition of SIT formation suppressed hemorrhage associated with both delayed reperfusion and delayed t-PA treatment. Furthermore, MMP activity, as well as MMP-9 antigen, localized within SIT. These findings suggest that hemorrhage associated with delayed recanalization by mechanical and t-PA treatment in ischemic stroke were induced through the activation of t-PA/plasmin/MMP-9 pathway initiated on SIT. In addition, it is suggested that hemorrhage associated with both delayed EVT and delayed t-PA therapy can be prevented by pharmacological inhibition of SIT formation.

## Data Availability

The original contributions presented in this study are included in the article/Supplementary Materials. Further inquiries can be directed to the corresponding author.

## Supplementary Materials

The following supporting information can be downloaded at: https://www.mdpi.com/article/10.3390/biomedicines14020308/s1.
