# Supplementary material for: Involvement of Secondary Induced Thrombus on Hemorrhage Induced by Both Delayed Recanalization and Delayed t-PA Treatment in Murine Ischemic Stroke Models
Source: Biomedicines. 2026 Jan 29;14(2):308. doi: 10.3390/biomedicines14020308 (PMC12938425; doi:10.3390/biomedicines14020308)
Supplement: Supplementary file 1 [file biomedicines-14-00308-s001.zip › biomedicines-4097164-supplementary.pdf]

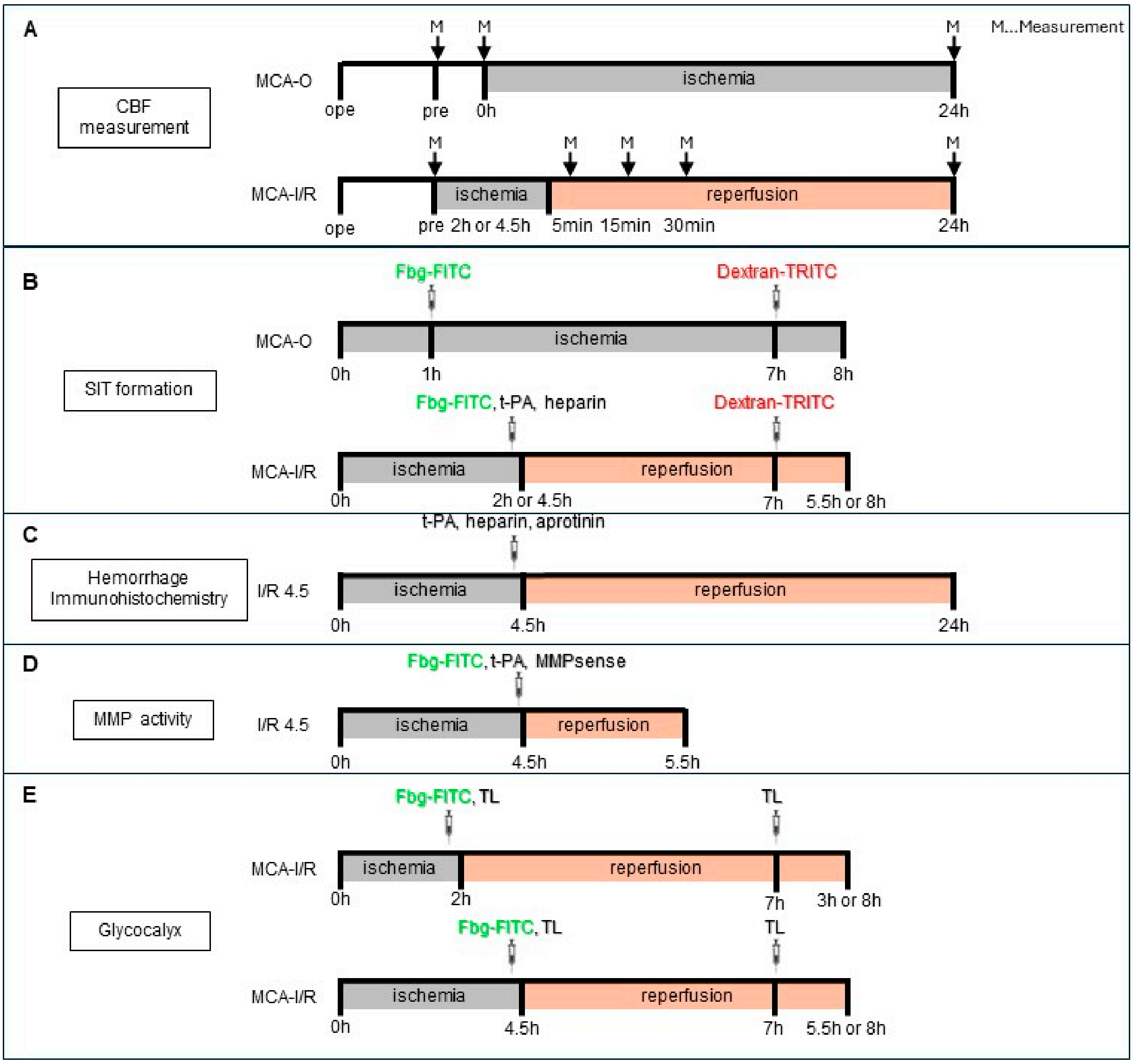

Supplemental Figure S1. Experimental setup.

Model, treatment, and time course for the analysis of cerebral blood flow CBF measurement (A), SIT formation (B), Hemorrhage and Immunohistochemistry (C), MMP activity (D), and Glycocalyx (E) are described.
